# Equine Asthma Does Not Affect Circulating Myostatin Concentrations in Horses

**DOI:** 10.3390/ani14050799

**Published:** 2024-03-04

**Authors:** Sylwester Kowalik, Maisie O’reilly, Artur Niedźwiedź, Witold Kędzierski

**Affiliations:** 1 Department of Animal Physiology, Faculty of Veterinary Medicine, University of Life Sciences in Lublin, ul. Akademicka 12, 20-033 Lublin, Poland; sylwester.kowalik@up.lublin.pl; 2 Department of Internal Medicine and Clinic of Diseases of Horses, Dogs and Cats, Faculty of Veterinary Medicine, Wroclaw University of Environmental and Life Sciences, Grunwaldzki Sq. 47, 50-366 Wrocław, Poland; 108883@student.upwr.edu.pl (M.O.); artur.niedzwiedz@upwr.edu.pl (A.N.); 3 Department of Biochemistry, Faculty of Veterinary Medicine, University of Life Sciences in Lublin, ul. Akademicka 12, 20-033 Lublin, Poland

**Keywords:** myostatin, horse, asthma, respiratory disorders, marker

## Abstract

**Simple Summary:**

The increase in the number of horses suffering from chronic respiratory diseases prompted us to undertake research on markers useful in the quick and accurate diagnosis of asthma in horses. Recent studies provide evidence for the usefulness of one myokine in the diagnosis of human asthma, namely, myostatin. Myostatin is an extracellular cytokine mostly expressed in skeletal muscles and known to play a crucial role in the negative regulation of muscle mass and function. Therefore, our aim was to compare the concentration of myostatin in the blood plasma of completely healthy and asthmatic horses. The research was carried out in two stages. In the first stage, horses with severe asthma were selected, and in the second stage, the myostatin level was determined in horses classified as asthmatic or healthy (controls). The experimental results did not provide a clear answer as to whether myostatin can be used as a reliable marker confirming the occurrence of equine asthma. Its level in horses suffering from asthma and healthy horses of similar age and use did not differ statistically, but was significantly higher compared to that in young race horses. Thus, further studies are needed to confirm our findings in a larger population of horses.

**Abstract:**

(1) Background: The number of horses suffering from chronic respiratory diseases, resembling human asthma, is increasing but there is still a lack of reliable and accurate methods to detect these disorders. Numerous studies have found elevated plasma concentrations of one of the myokines, namely, myostatin (MSTN), in people suffering from severe asthma. MSTN normally inhibits myoblast proliferation and differentiation through autocrine or paracrine signals. Therefore, given the pathogenesis of asthma, we hypothesize that MSTN could be a useful biomarker of equine asthma. Thus, this study aimed to compare the concentration of MSTN in the blood plasma of fully healthy and asthmatic horses. (2) Methods: A total of 61 horses were clinically examined to confirm or exclude the occurrence of equine asthma, including bronchoalveolar lavage (BAL) fluid cytology performed on 49 horses. This study included three groups of horses, two of which were clinically healthy, and one of which was asthmatic. (3) Results: The mean circulatory MSTN concentration determined using the ELISA method in asthmatic horses was significantly higher than that in clinically healthy young Thoroughbred racehorses (*p* < 0.05), but it did not differ as compared to the group of healthy, adult leisure horses. (4) Conclusions: The obtained results did not unambiguously support our original hypothesis that MSTM may be a reliable marker for the early diagnosis of equine asthma. To the best of the authors’ knowledge, this is the first study to analyze the plasma MSTN concentration in equine asthma patients, and therefore further studies are needed to confirm our novel findings.

## 1. Introduction

According to field reports, one of the largest problems in modern equine medicine is equine asthma (EA) syndrome, that may affect up to 80% of adult horses, depending on the population and geographic region [1]. This disease causes significant losses in the use of horses in various sports and work disciplines [2]. Nowadays, the diagnosis of EA is most often based on clinical signs, examination results, and treatment response due to the lack of conclusive and relatively quick diagnostic tests [3,4,5]. Recently, in human medicine, asthma and asthma-like diseases have been recognized as complex and heterogeneous disorders characterized by airway obstruction and chronic inflammation, resulting in progressive airway muscle damage [6]. Physiologically, muscle tissue is distinguished by a relatively high plasticity, leading in extreme cases to the hypertrophy or atrophy of myocytes [7]. The proper mass of both smooth and skeletal muscle is controlled by the endocrine system, and myokines play a key role in this regulation [8,9,10]. One of these is myostatin (MSTN), formerly known as growth and differentiation factor 8 (GDF8), a member of the transforming growth factor-β superfamily [11,12]. The MSTN gene, located on equine chromosome 18 (ECA 18), is expressed in skeletal muscle tissue and acts as a negative regulator of skeletal muscle mass [13]. The loss of MSTN gene function leads to a dramatic and widespread increase in skeletal muscle mass, while its overexpression or systemic administration causes muscle atrophy [7,14,15]. Although the regulation of muscle weight is a process in which MSTN plays a central role, the mechanism of its action and signaling cascades are not fully understood. So far, it is known that MSTN can act through the insulin-like growth factor 1 (IGF-1)/phosphatidylinositol 3-kinase (PI3K)/Akt pathway largely responsible for the increase in muscle protein synthesis [16,17]. In other words, the myostatin-induced repression of cell proliferation is inhibited by insulin-like growth factor 1 (IGF-1) or Akt activation while the attenuation of IGF-1 signaling causes myoblasts to undergo apoptosis in response to myostatin action [18]. Therefore, MSTN negatively affects muscle growth by inhibiting myoblast proliferation and differentiation [19,20]. Findings in humans indicate increased MSTN expression in subjects with primary or secondary cachexia, sarcopenia, heart failure, HIV, and, finally, pulmonary diseases [21,22,23,24,25,26]. The recent study completed by Tan et al. [27] showed that the overexpression of MSTN also stimulates apoptosis in airway smooth muscle cells, which ultimately leads to chronic obstructive pulmonary disease (COPD) and asthma. The associations of MSTN with pulmonary disease mentioned above are relevant to human medicine, but there are no relevant reports in horses. Given the pathogenesis of asthma, MSTN could be also a useful biomarker of asthma in horses; however, the skeletal muscle mass and/or training status could also be factors influencing plasma MSTN concentration in horses [9,19].

In view of the above, this study was designed to test the hypothesis that plasma myostatin concentration may be a reliable biochemical marker of EA. Thus, the concentration of MSTN in the blood plasma of healthy horses and horses suffering from asthma was compared, using two control groups of healthy horses different in age and physical performance.

## 2. Materials and Methods

The whole study was carried out in two stages. In the first stage of the research, field studies were carried out to select and, on this basis, create groups of horses suffering from asthma and healthy control horses. In the second stage, circulatory MSTN was determined in horses classified as asthmatic or healthy.

### 2.1. Horses

This study was carried out on 61 horses acquired from a private source, representing different breeds and types of usage. They were 21 Konik Polski horses and 28 Polish half-bred horses used as leisure or pet horses, and 12 Thoroughbred horses trained for races. Some Konik Polski and Polish half-bred horses showed signs of exercise intolerance and airway disorders, and were treated for pulmonary diseases prior to the study. Thoroughbred horses were healthy, sound, and free of clinical disease at the time of the study. They did not show any signs of poor performance, exercise intolerance, or airway disorders such as acute or chronic inflammation or cough syndrome.

The leisure and pet horses (Konik Polski and Polish half-bred horses) were kept in spacious, straw-bedded individual boxes with free access to hay, water, and mineral salt blocks. In the daytime, when they were not used for riding, the horses were grazed on a pasture that was based upon a mixture of meadow grass. In addition, oats and commercial concentrate were given to the horses three times a day (06.00 am, 12.00 am, and 06.00 pm), in accordance with their individual nutritional requirements. Because the horses were kept in different stables under different environmental conditions, the microclimate parameters in the stables could slightly differ. Nevertheless, in each of the stables, a gravity method of air exchange was provided by creating air passages between the ceiling vents, windows, and partially open doors. As a result, stable air conditions did not deviated from the accepted and prevailing norm. 

The Konik Polski and Polish half-bred horses, which did not show any health problems, were moderately work-loaded as horses intended for recreational riding in their home equestrian centers. They were routinely exercised five to six times per week, and the daily workload consisted of walk, trot, and sometimes canter at 4–5 m/s under the rider. The daily workload for each horse was established by an experienced riding instructor. 

All 49 Konik Polski horses and Polish half-bred horses mentioned above were subjected to tracheobronchial endoscopic examination, under the protocol described below, to absolutely confirm or exclude the occurrence of asthma in the horses for which an MSTN analysis of blood plasma would be conducted. 

The race Thoroughbred horses were individually housed in spacious, straw-bedded boxes with free access to water and mineral salt blocks. Following their needs (i.e., depending on workload and body condition), each horse received an individually calculated ration of hay, oats, and commercial supplements formulated for equine athletes distributed three times per day (06.00 am, 12.00 am, and 06.00 pm). The dustiness within stables was kept to a minimum by the good flow of the air. For this purpose, a system combining gravity and forced ventilation was set up in the stable. This double ventilation was provided by the installation of ceiling fans, as well as leaving an air channel between the roof and the walls, and by built-in and partially opening windows. In addition, the top of the stable door was partially open all the time to maintain levels of environmental dust at a minimum. 

The group of race Thoroughbred horses were routinely exercised six times per week on a professional race track. It was their second training season, and all training procedures were carried out under the care of one professional trainer. The typical daily training routine consisted of the following: 10 min of walking as a warm-up, 10 min of trot or canter, and galloping over the distance of 1200 m at a speed between 6 and 12 m/s, according to horse performance and the trainer’s instructions. Every training session was completed by putting the horses on an automatic horse walker for 30 min to cool them down. The Thoroughbred horses took part in official national races during the current race season. Therefore, bearing in mind their high race performance, there were no grounds for medical referral for a bronchoscopic examination of their airways for asthma; hence, such an examination was not performed.

### 2.2. Classification of Horses into Experimental Groups

EA was confirmed or excluded in accordance with the adopted four-point plan including (1) the clinical history of the horses; (2) clinical examinations performed at rest, during, and after exercise, (3) tracheobronchial endoscopy for an analysis of the airway mucosa and the quantity and quality of bronchial mucus; and (4) BAL cytology and pro-inflammatory cell counting.

Ultimately, based on the obtained results of the clinical, endoscopic, and BAL examination, the horses were classified for MSTN analysis as asthmatic (Astm) or clinically healthy leisure (Lr) horses.

The Astm group consisted of 15 horses: 4 Konik Polski horses and 11 Polish half-breed horses (7 geldings and 8 mares). The mean age of the horses from this group amounted to 15 years (from 7 to 23 years). The disease history of these horses was known—they were moderately loaded with work as recreational horses before the onset of the first asthma symptoms. As the symptoms progressed and the clinical picture of the disease worsened, the horses were gradually withdrawn from daily work. Finally, they were completely excluded from exercise due to severe asthma symptoms, and for at least 6 months, they were not used as riding horses. Therefore, all horses in this group were treated for respiratory symptoms before the study. They received corticosteroids and long-acting bronchodilators (β2-adrenergic receptor agonists), but all medications were discontinued 4 weeks before the study. 

The Lr group consisted of ten clinically healthy horses, including seven Polish Konik horses and three Polish Half-Breed horses, with a mean age of 16.5 years (from 10 to 23 years; six geldings and four mares). All the Lr horses were moderately work-loaded as horses intended for recreational riding in their home equestrian centers. 

Thoroughbred horses (Th) were included in the study as a second control group of healthy animals. These were individuals aged 3–4 years, with an equal number of stallions and mares.

### 2.3. Body Condition Score

For each horse classified for the MSTN analysis, the body condition score (BCS) was determined with the use of the rating system, ranging from 1 to 9 [28].

### 2.4. Endoscopic Examination and Bronchoalveolar Lavage Fluid Collection and Analysis

An endoscopy of the airways and bronchoalveolar lavage were performed in horses sedated with 0.01 mg/kg of detomidine (Domosedan, Orion Corporation, Espoo, Finland) and 0.01 mg/kg of butorphanol (Morphasol, aniMedica GmBH, Senden-Bösensell, Germany). A 1.8 m long endoscope was passed through the nasal passage into the trachea (Karl Storz GmbH, Tuttlingen, Germany). Mucus accumulation in the airway lumen was clinically graded on a 6-point scale, where 0—no visible mucus; 1—little, single to multiple small blobs of mucus; 2—moderate, larger, but not confluent blobs; 3—marked, confluent, or stream-forming mucus;, 4—large, pool-forming mucus; and 5—extreme, profuse amount of mucus [29]. Bronchoalveolar lavage was carried out by instilling 400 mL of sterile saline (0.9% NaCl) at body temperature through the endoscope working channel into the bronchus using successive 60 mL boluses. The bronchoalveolar lavage (BAL) fluid was then re-aspirated through gentle suction using a 60 mL syringe until no further fluid could be obtained. The amount of recovered fluid was recorded and the BAL for each individual horse was pooled in a sterile specimen cup, placed on ice, and processed within 2 h from collection. In order to carry out the cytologic examination of the BAL, a 10 mL aliquot was centrifuged at 300× *g* for 10 min using a centrifuge (Beckman Coulter Allegra x-22; Beckman Coulter Inc., Brea, CA, USA), and the smear of the sediment was stained with May–Grunwald–Giemsa (MGG) stain. A 400-cell leukocyte differential count was performed. Epithelial cells were not included in the differential count.

### 2.5. Blood Collection and Analysis 

This study protocol was reviewed and approved by the Local Ethic Review Committee for animal experiments (38/2023). The owners of the horses participating in the study were aware of the purpose of the study, the risks and benefits, and gave their oral consent to blood sampling.

Blood samples were collected at the turn of April and May. On the test day, all horses had undergone thorough clinical examinations including clinical history and physical examinations. All horses were examined by the same veterinarian, a specialist in equine medicine. 

The study was conducted in spring, before training intensification and the grazing period, to avoid training effects. Sampling took place in the early morning, before feeding and any horse activities. For each subject, blood sample was drawn by vena jugularis externa venipuncture using BD Vacuette vacuum system into the tubes containing EDTA and aprotinin (Becton Dickinson, New Tork, NY, USA, No. ref.: 454261) to preserve the integrity of the sample through proteolysis inhibition. Blood samples were centrifuged at 300× *g* for 15 min at 4.0 °C. The obtained plasma samples were frozen at −20 °C until further analysis. Total MSTN levels were measured using competitive antibody sandwich enzyme-linked immunosorbent assay (ELISA) kits (GDF-8/Myostatin, R&D System Inc., Minneapolis, MN, USA), based on antibodies raised against recombinant MSTN (GDF-8), validated previously [30]. The assay was performed in duplicate, according to the manufacturer’s specifications. The intra- and inter-assay CVs were <5.4% and <6.0%, respectively. The absorbance was measured with a Multiscan reader (Labsystem, Helsinki, Finland), using a GENESIS V 3.00 software program (VWR International Ltd., Lutterworth, UK). 

### 2.6. Statistical Analysis 

The data were analyzed with regard to the normal distribution using Shapiro–Wilk test. The result of the test confirmed the normality of the data distribution. Then, an analysis of variance (ANOVA) was used to analyze the influence of the group factor (the experimental one with pulmonary asthma and the two control groups). Next, a post hoc comparison of the means was performed using Tukey’s test. The level of statistical significance was set at *p* ≤ 0.05. Continuous data are presented as means ± standard deviation (SD). Statistical analysis was performed using the GraphPad Prism 6.0 software package (GraphPad Software Inc., San Diego, CA, USA).

## 3. Results

The BAL results and the percentage number of non-degenerate neutrophils calculated in BAL smears of examined horses are presented in Table 1. On the basis of the percentage amount of non-degenerated neutrophils in the BAL analysis, horses were classified as healthy (<5%) or asthmatic (>20%). Horses in which the percentage of neutrophils amounted to 5–15% (indirect results) were not included in the MSTN analyses.

Ten Lr horses were classified into the group of healthy animals in which asthma was excluded based on the tests performed. Based on the same tests, 15 horses were classified as asthmatic (Astm). Due to questionable asthma tests results, 24 horses were excluded from further study to determine the level of circulatory MSTN. Additionally, 12 racing horses (Th) were included in the study as the second control group, in which there was no suspicion of asthma due to their young age and impeccable racing career spanning at least one year. 

The detailed results of BCS estimation in the tested horses are presented in Table 2. The data show that the mean BCS in the group of Th was significantly lower than that in the other groups.

**Figure 1:**
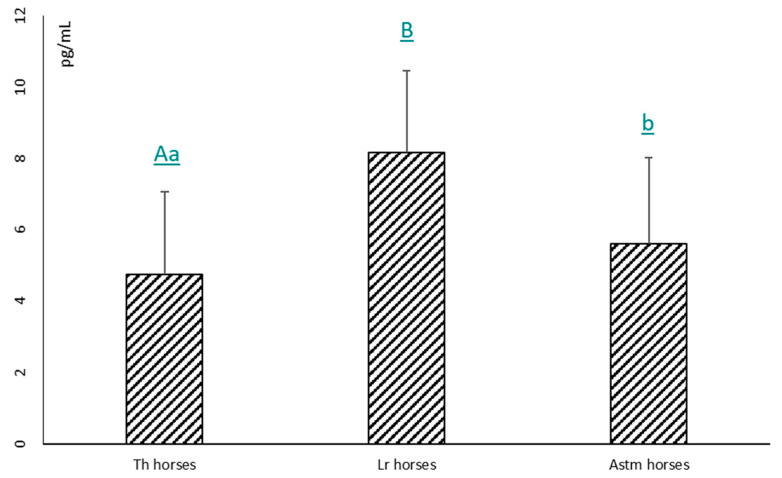
The mean values of MSTN in the blood plasma of the investigated horses ± SD. A, B—different letters indicate significant differences at *p* ≤ 0.01; a, b—different letters indicate significant differences at *p* ≤ 0.05.

The mean plasma MSTN concentrations in the studied groups of horses are shown in Figure 1. The results of the study revealed that Th horses had the lowest plasmatic MSTN concentration, in comparison to the others. As a consequence, both Astm horses and Lr horses had significantly higher myostatin levels than Th horses (*p* < 0.001 and *p* < 0.05, respectively). Differences in plasma myostatin levels between the Lr group and Astm group (8.16 ± 2.3 vs. 5.61 ± 2.4 pg/dL, respectively) were not statistically significantly different (*p* > 0.05).

## 4. Discussion

A few recent reports from human medicine prompted us to undertake research on MSTN in horses and its potential use as a marker of asthmatic conditions in horses. Specifically, research conducted on COPD human patients has shown that an increased level of circulatory MSTN leads to muscle cell atrophy [31]. In addition, Hayot et al. [32] indicated that the observed increase in the plasma concentration of MSTN was correlated with chronic lung diseases causing primary hypoxemia and secondary muscle cell hypoxia. Similarly, Ju et al. [33] found that COPD patients with *cor pulmonale* had higher levels of circulating MSTN than controls. Moreover, COPD patients without any other complications had significantly higher levels of MSTN than healthy controls. Therefore, it can be assumed that the increase in the MSTN concentration progresses with the increasing limitation of airflow through the respiratory tract and subsequent chronic hypoxemia, which may be the cause of muscle cell damage. It should be mentioned that already in 1998, Bernard et al. [34] noticed the existence of a relationship between chronic hypoxia and muscle mass and the force of muscle contraction. Specifically, in patients with COPD, the cross-sectional area of muscle cells and the force developed by them were significantly lower [22,23,24,26]. To date, at least two of the most common lung tissue diseases associated with MSTN expression have been identified, namely, COPD and asthma [33,35]. A clinical approach to differentiate between COPD and asthma in humans is difficult due to their many related features. As a result, asthma and COPD are often referred to as the asthma-COPD overlap syndrome (ACOS) [36,37]. 

The pathophysiology of asthma in humans and severe equine asthma in horses highlights a key difference in the inflammatory response and the type of cells involved in each species [38]. In human asthma, the inflammation of the airways is predominantly eosinophilic, involving eosinophils (a type of white blood cell) along with other inflammatory cells like mast cells and T lymphocytes. This eosinophilic inflammation leads to airway hyperresponsiveness, mucus production, and bronchoconstriction, causing the typical symptoms of asthma such as wheezing, shortness of breath, and coughing [39,40]. In contrast, severe equine asthma involves a more neutrophilic inflammatory response, with neutrophils (another type of white blood cell) being the predominant cell type involved in the airway inflammation [41,42]. In horses with severe equine asthma, the airway inflammation leads to excessive mucus production, airway obstruction, and difficulty breathing, which can be particularly evident during episodes of exacerbation triggered by environmental factors like dust and mold [41,43]. Therefore, currently, the term “equine asthma” is used to describe horses with chronic respiratory symptoms ranging from mild to severe, which were previously called inflammatory airway disease (IAD), recurrent airway obstruction (RAO), and summer pasture-associated obstructive airway disease [38,44]. 

To the best of our knowledge, this is the first study to compare plasma myostatin concentrations in healthy and asthmatic horses. We hypothesized that the level of MSTN in horses affected by asthma should be high, due to the presence of local hypoxia in airway smooth muscle (ASM); hence, we investigated whether MSTN could be a useful marker of EA. Because the MSTN concentration did not differ in the asthmatic horses as compared to healthy adult horses of the same breeds and type of use, the obtained results did not confirm our initial hypothesis. When planning this study, we did not expect comparable results of MSTN concentrations in the blood plasma of these groups of horses. These results were even more surprising because the previously mentioned data from the literature indicate that the level of MSTN in patients with asthma should be higher than that in healthy subjects. On the other hand, the plasma MSTN concentration in adult asthmatic horses was higher than that in healthy young race horses. Although this result was not surprising to us, we would like to discuss this phenomenon, as we believe that several methodological considerations should be clarified.

In the present study, circulating MSTM levels were not statistically different in asthmatic horses compared to recreational horses (5.61 vs. 8.16 pg/mL, *p* > 0.05), despite the fact that the horses from these groups were similar in terms of breed, age, and usage. Therefore, the possible reason for this fact may result from the classification of animals suffering from various degrees of asthma severity to the group of asthmatic horses, the only common denominator of classification of which was the clinical picture and BAL results, but not the degree of asthma severity. Furthermore, it should be noted that in patients receiving corticosteroids and/or β2-adrenergic agonists as a symptomatic treatment of pulmonary obstructive diseases, ASM remodeling and/or damage processes are inhibited [34,45]. This may also explain why MSTN levels were not as high as expected in our asthmatic horses. In other words, if corticosteroids or long-acting β2-adrenoceptor agonists were used before the horses were enrolled in the study, they probably inhibited the progression of asthma, including the remodeling of the smooth muscle cells of the bronchial tree and the development of subsequent local hypoxia [46]. As a result, the plasma MSTN levels did not increase dramatically in comparison to the controls.

A possible reason for differences in MSTN concentration in humans with asthma and horses with severe equine asthma could be related to the role of MSTN in muscle metabolism and the distinct physiological responses to chronic respiratory conditions in each species. MSTN is a protein that inhibits muscle growth, playing a crucial role in regulating muscle mass. In humans with asthma, especially those with severe or poorly controlled asthma, physical activity levels can be lower due to respiratory symptoms and limitations [47,48]. Reduced physical activity could lead to decreased muscle mass or changes in muscle composition, potentially influencing MSTN levels as the body tries to regulate muscle growth and metabolism in response to decreased demand. In contrast, horses with severe equine asthma experience respiratory distress that can significantly affect their performance and physical activity levels [43]. However, the relationship between MSTN concentration and severe equine asthma may be influenced by different factors compared to humans. For instance, the management strategies for horses with this condition, including changes in environment and exercise routines, might not directly correlate with the same physiological or metabolic adaptations seen in humans [49,50]. Furthermore, the role of MSTN in horses might be more complex due to their large muscle mass and the different demands placed on their musculoskeletal system. Therefore, differences in MSTN concentration could reflect species-specific responses to chronic respiratory conditions, influenced by factors such as physical activity levels, muscle metabolism, and the systemic effects of chronic inflammation associated with asthma and severe equine asthma [51,52]. 

One of the major limitations in the general study of asthma in horses is that peripheral tissue samples cannot be collected without invasive methods such as, e.g., endobronchial biopsy. Although the material collected in this way provides reliable information on the primary structure and status of the lung tissue, this method cannot be used without the prior clinical preparation of the animals. Therefore, clinically speaking, it is important to identify a suitable marker that would indicate EA. Nevertheless, in our research, the used control groups of healthy horses differed in plasma MSTN concentration. Statistically higher values of MSTN were found in Lr horses than in young Th racehorses. In previous studies, it was found that both intense and endurance exercises significantly stimulate an increase in the plasmatic MSTN level in horses [30]. This effect of exercise was also observed in humans [53,54]. Moreover, when we analyzed the baseline MSTN circulatory concentration, we found higher values in Arabian racing horses aged 7 to 12 years than in 3-year-old horses starting racing training [30]. We believe that this phenomenon is caused by the fact that young horses during training are in the phase of skeletal muscle growth and development, whereas in older horses not used intensively, muscle growth is under greater MSTN control. Thus, its level is related to the age of the horses and the stage of their training. It is known that in humans the effect of MSTN on muscle tissue is related to age [55,56]. 

Furthermore, based on analyzing the BCS, a different explanation for the higher MSTN levels in the Lr and Astm groups than in Th comes to mind: the key may be that older horses tend to have more body fat compared to young athletic horses. It has been previously confirmed that MSTN can be produced by adipose tissue [57]. It was also stated that obese horses had higher circulating MSTN levels than lean individuals [10]. Further research is needed to define the adipose-dependent physiological pathways of MSTN in healthy and asthmatic horses. We believe, however, that a better understanding of the endocrine and molecular mechanisms of EA mediated by the myostatin pathway could be a significant aid in the monitoring and/or treatment of asthmatic patients.

## 5. Conclusions

In conclusion, based on the presented preliminary study, it is not possible to conclusively state whether MSTM may be a reliable marker for the diagnosis of asthma in horses. Probably, a different breed, age, and type of physical activity are factors that affect plasma myostatin levels to a great extent than the presence of asthma in horses. Nevertheless, more detailed research is needed to confirm these findings in a larger population of horses.

## Figures and Tables

**Table 1:** Results of equine asthma (EA) diagnostics in the studied horses.

	Recreational Horses (Lr)	Other Horses (Excluded)	Asthmatic Horses (Astm)
Breed	Konik Polski (n = 7) Polish Half-Bred Horse (n = 3)	Konik Polski (n = 10) Polish Half-Bred Horse (n = 14)	Konik Polski (n = 4) Polish Half-Bred Horse (n = 11)
Clinical symptoms	None	Occasional cough with periods of no coughing Flares during inspiration Slight flattening of ventral flank	Very frequent cough Flares in inspiration and expiration (no movement can be seen) Obvious abdominal lift and ’’heave line’’ extending beyond halfway between the cubital joint and tuber coxae
Mucus accumulation score and its colour *	0 clear	1–2 yellow	3–5 yellow
% of non-degenerated neutrophils in BAL	<5	5–15	>20
% of eosinophils	0–0.2	0–0.5	0–0
% of mast cells	0.5–2.0	0.5–3.0	1.0–3.0
Presence of Curschmann‘s spirals	No	Occasionally	Yes

* mucus scoring system according to Gerber et al., 2004 [29].

**Table 2:** Demographic summary of the studied horses divided into three experimental groups.

	Racehorses (Th)	Recreational Horses (Lr)	Asthmatic Horses (Astm)
Breed	Thoroughbred horse (n = 12)	Konik Polski (n = 7) Polish half-bred horse (n = 3)	Konik Polski (n = 4) Polish half-bred horse (n = 11)
Total numbers of individuals in group	12	10	15
Mean age (years)	3.5 (3—4) ^a^	16.5 (10—23) ^b^	15 (7–23) ^b^
Intensity and type of usage	Intensive, race horses	Moderate, recreational horses	Moderate, recreational horses
Mean BCS (Body Condition Score)	4.2 ^a^	6.3 ^b^	6.1 ^b^

^a, b^—different letters indicate significant differences at *p* ≤ 0.05.

## Data Availability

The data presented in this study are available on request from the corresponding author.
